# Genetic Insights into Colorectal Cancer: Evaluating PI3K/AKT Signaling Pathway Genes Expression

**DOI:** 10.3390/ijms25115806

**Published:** 2024-05-27

**Authors:** Rafał Świechowski, Jacek Pietrzak, Agnieszka Wosiak, Michał Mik, Ewa Balcerczak

**Affiliations:** 1 Department of Pharmaceutical Biochemistry and Molecular Diagnostics, Medical University of Lodz, Muszynskiego 1, 90-151 Lodz, Poland; 2 BRaIn Laboratories, Medical University of Lodz, Czechoslowacka 4, 92-216 Lodz, Poland; 3 Department of General and Colorectal Surgery, Medical University of Lodz, ul. Zeromskiego 113, 90-549 Lodz, Poland

**Keywords:** colorectal cancer, PI3K/AKT pathway, gene expression, *PIK3CA*, *AKT1*

## Abstract

The PI3K/AKT pathway plays a pivotal role in cellular processes, and its dysregulation is implicated in various cancers, including colorectal cancer. The present study correlates the expression levels of critical genes (*PIK3CA*, *PTEN*, *AKT1*, *FOXO1*, and *FRAP*) in 60 tumor tissues with clinicopathological and demographic characteristics. The results indicate age-related variation in *FOXO1* gene expression, with higher levels observed in patients aged 68 and above. In addition, tumors originating from the rectum exhibit higher *FOXO1* expression compared to colon tumors, suggesting region-specific differences in expression. The results also identify the potential correlation between *PTEN*, *PIK3CA* gene expression, and parameters such as tumor grade and neuroinvasion. The bioinformatic comparative analysis found that *PTEN* and *FOXO1* expressions were downregulated in colorectal cancer tissue compared to normal colon tissue. Relapse-free survival analysis based on gene expression identified significant correlations, highlighting *PTEN* and *FRAP* as potential indicators of favorable outcomes. Our findings provide a deeper understanding of the role of the PI3K/AKT pathway in colorectal cancer and the importance of understanding the molecular basis of colorectal cancer development and progression.

## 1. Introduction

Transforming growth factor β (TGF-β) is a pleiotropic cytokine that performs a range of principal functions in the body, including cell growth and differentiation [1]. TGF-β may act as a suppressor or promoter of carcinogenesis, depending on the stage and type of cancer. Regardless of the canonical pathway involving SMAD proteins, the TGF-β cytokine can interact with the PI3K/AKT pathway [2], also known as the phosphoinositide 3-kinase/protein kinase B pathway, which is a critical cellular signaling cascade that plays a crucial role in regulating cell growth, survival, apoptosis, migration, and metabolism [3]. Although the pathway is essential for normal cell function, it can also contribute to the development of diseases, notably cancer, when dysregulated.

The key components of the pathway are phosphatidylinositol 3-kinase (PI3K), receptor tyrosine kinases (RTKs), phosphatase and tensin homolog deleted on chromosome ten (PTEN), protein kinase B (AKT), mammalian target of rapamycin (mTOR), and FOXO transcription factors [2,4]. Upon activation by RTKs or other cell surface receptors, PI3K phosphorylates phosphatidylinositol 4,5-bisphosphate (PIP2) to generate phosphatidylinositol 3,4,5-trisphosphate (PIP3). PIP3 serves as a second messenger in the pathway. PIP3 can be dephosphorylated back to PIP2 by PTEN which acts as a tumor suppressor by limiting PI3K/AKT signaling [5]. When PIP3 levels increase due to PI3K activation, AKT is recruited to the plasma membrane.

While AKT kinase activation is not sufficient to start oncogenesis, it can influence cancer progression. AKT regulates the activity of subsequent proteins responsible for the main functions of the PI3K/AKT signaling pathway. It phosphorylates mTOR kinase, which is part of the TORC1 protein complex that regulates anabolic processes such as protein synthesis and ribosome biogenesis, and TORC2, which is responsible for cell proliferation and organization of the cytoskeleton [6]. AKT kinase also regulates the expression of proteins from the FOXO family, which are some of the most important effectors of the PI3K/AKT pathway; these regulate the expression of genes involved in apoptosis, cell cycle, and DNA repair. Their function is negated by phosphorylation [6,7].

The increased activity of the PI3K/AKT pathway is associated with inter alia colorectal cancer (CRC) [8]. According to data from GLOBOCAN 2020, CRC is one of the most widely-diagnosed cancers worldwide: in 2020, it was estimated to be the third most common cancer in men and the second in women. Its incidence is highest in developed countries [9]. However, survival rates for CRC have been improving, particularly in countries with well-established screening programs and advanced treatment options, and the chances of survival are significantly improved by early-stage diagnosis and timely treatment. The five-year survival rate has now exceeded 60% in numerous European countries; however, this is strongly dependent on the stage at which the disease is diagnosed. For instance, patients diagnosed at stage I have a survival rate of approximately 90%, but this rate falls to around 10% at stage IV [9,10]. Nevertheless, the newest report from GLOBOCAN showed that over 930,000 deaths were caused by CRC worldwide in 2020, which constituted about 10% of all cancer-related deaths [11]. These data underline the need to continue searching for new treatment schemes and inventing new markers that will play a crucial role in prevention programs and in selecting the appropriate type of pharmacological treatment.

The aim of the study was to evaluate the expression of five key genes encoding the proteins included in the PI3K/AKT signaling pathway, namely *PIK3CA* (encoding the catalytic subunit of PI3K), *PTEN*, *AKT1* (encoding AKT kinase), *FOXO1*, and *FRAP* (encoding mTOR kinase), in relation to two reference genes: *GAPDH* and *ACTB* in patients with CRC. The obtained results were correlated with the following clinicopathological and demographic characteristics: age, sex, tumor location, histological grade, disease advancement according to TNM classification (The American Joint Committee on Cancer; AJCC), number of affected lymph nodes, presence of tumor angiogenesis and neuroinvasion. In the next step, a bioinformatics analysis was carried out, which took into account the molecular subtype of the CRC and the presence of *PIK3CA* and *PTEN* gene mutation. Using the data contained in the databases, the expression levels of the analyzed genes were compared between tumor tissue and normal tissue, and survival curves were created in regard to the gene expression level.

## 2. Results

### 2.1. The Association between Gene Expression and the Sex and Age of Patients

**Figure 1:**
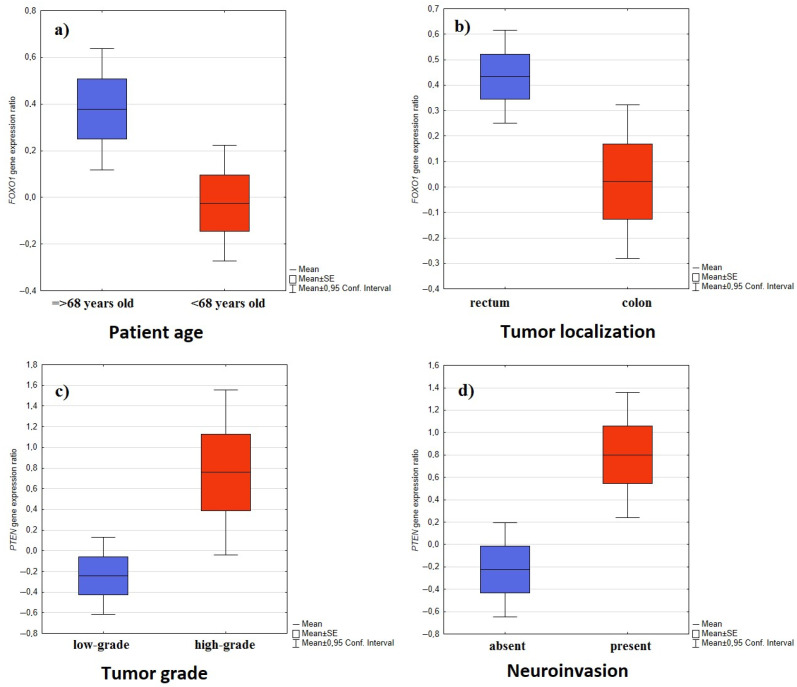
*FOXO1* and *PTEN* gene expression values after BOX-COX transformation with regard to various clinicopathological and demographic features; (**a**) *FOXO1* gene expression with regard to patient age; (**b**) *FOXO1* gene expression with regard to tumor localization; (**c**) *PTEN* gene expression with regard to tumor grade; (**d**) *PTEN* gene expression with regard to the presence of neuroinvasion.

No significant differences were noted in the expressions of *PIK3CA*, *PTEN*, *AKT1*, *FOXO1*, or *FRAP* genes with regard to sex. In addition, no statistically significant correlation based on correlation matrices was found between patient age and gene expression. The patients were divided into two groups based on the median age (68 years); *FOXO1* gene expression was found to be significantly higher in the group of patients aged 68 and above (*p* = 0.0268) compared to the younger group (Figure 1).

### 2.2. The Association between Gene Expression and the Cancer Stage According to the TNM Scale (AJCC)

In the first analysis, no statistically significant differences in gene expression were observed with regard to tumor size (T). In addition, no statistically significant differences in expression were noted with regard to the number of involved lymph nodes (N) when divided into three groups (N0, N1, and N2) (classification according to the TNM) or when divided into two groups (N0 vs. N1, N2). Furthermore, no differences in gene expression were observed based on the presence of distant metastases (M0 vs. M1). Finally, the patients were classified into a 4-stage scale of disease progression according to AJCC (I–IV stage); again, no significant differences in gene expressions were observed with regard to the cancer stage.

### 2.3. The Association between Gene Expression and Tumor Localization, Tumor Grading, Presence of Angioinvasion, and Neuroinvasion

Firstly, the patients were divided into two groups with regard to tumor location: those with tumors located in the colon and those with tumors located in the rectum. No significant differences in expression were found between the locations, apart from the *FOXO1* gene, which was more strongly expressed in the rectum than the colon (*p* = 0.0223) (Figure 1).

In addition, high-grade tumors demonstrated higher *PTEN* expression than low-grade tumors (*p* = 0.0308) (Figure 1). However, no statistically significant relationship was found for the remaining analyzed genes regarding tumor grade. The only exception was the *PIK3CA* gene, where higher expression was confirmed in women with low-grade tumors compared to high-grade tumors (*p* = 0.0401). However, after considering the Benjamini-Hochbergcorrection, these two dependencies lost statistical significance.

No significant differences in gene expression were observed with regard to angioinvasion. However, significantly higher *PTEN* expression was observed in cases with neuroinvasion (*p* = 0.0008) (Figure 1), both in the male group (*p* = 0.0080) and the female group (*p* = 0.0484).

### 2.4. Comparison of PIK3CA, PTEN, AKT1, FRAP, FOXO1 Gene Expression and Protein Levels between Tumor Tissue from Patients with Colorectal Cancer and Normal Tissue

**Figure 2:**
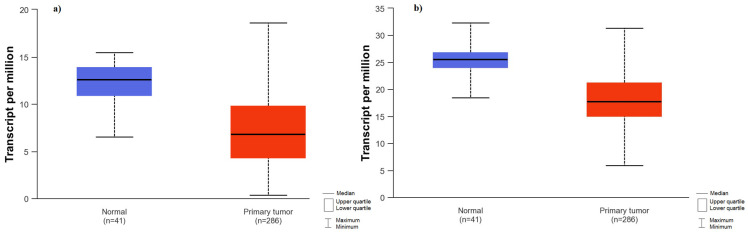
Comparison of gene expression between colon tumor tissue and normal tissue; (**a**) *FOXO1* gene expression in colon cancer tissue and normal tissue; (**b**) *PTEN* gene expression in colon cancer tissue and normal tissue.

**Figure 3:**
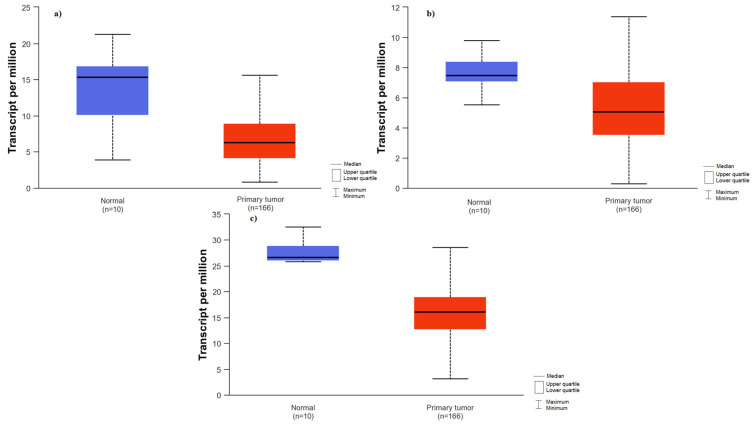
Comparison of gene expression between rectal tumor tissue and normal tissue; (**a**) *FOXO1* gene expression in rectal cancer tissue and normal tissue; (**b**) *PIK3CA* gene expression in rectal cancer tissue and normal tissue; (**c**) *PTEN* gene expression in rectal cancer tissue and normal tissue.

The expression of the analyzed genes was compared between normal colon and rectal tissue and tumor tissue based on the data available on the UALCAN platform. Firstly, an analysis was performed for colon adenocarcinoma. Significantly higher expression of *PTEN* (*p* < 0.0001) and *FOXO1* (*p* < 0.0001) were observed in normal tissue compared to tumor tissue (Figure 2). No significant differences were found for the rest of the analyzed genes. In the rectum, the *PTEN* gene (*p* < 0.0001) and *FOXO1* gene (*p* = 0.0099) also demonstrated higher expression in normal tissue compared to tumors. Additionally, higher *PIK3CA* gene expression (*p* = 0.0109) was noted in normal tissue compared to rectal tumors (Figure 3).

**Figure 4:**
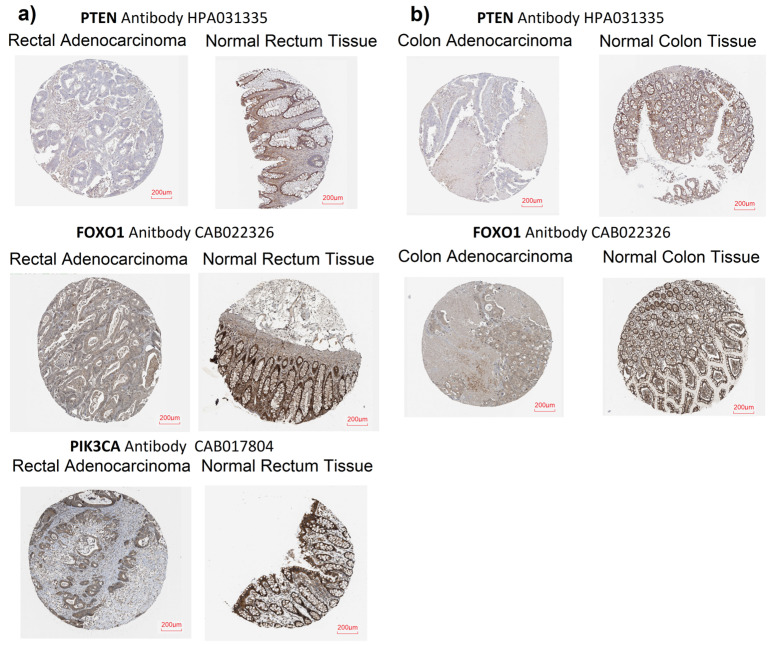
Comparison of protein expression level between tumor tissue and normal tissue; (**a**) PTEN, FOXO1, and PIK3CA protein expression in rectal adenocarcinoma and normal rectal tissue (Human Protein Atlas); (**b**) PTEN and FOXO1 protein expression in colon adenocarcinoma and normal colon tissue (Human Protein Atlas).

Additionally, after assessing the mRNA levels of selected genes, we compared the PIK3CA, PTEN, and FOXO1 protein expression between cancer and normal tissue using images from the Human Protein Atlas. We found that PTEN protein was not observed or the expression was low in colorectal cancer tissue, whereas medium expression was detected in both rectal and colon normal tissue. FOXO1 protein was found to be low/medium expressed in cancer tissue, while medium expression was noticed in normal colon and rectal tissue. Higher PIK3CA protein expression was observed in normal rectal tissue than in colorectal cancer tissue (Figure 4).

### 2.5. The Association between PIK3CA, PTEN, AKT1, FRAP, FOXO1 Gene Expressions and Presence of PIK3CA, PTEN Gene Mutation

**Figure 5:**
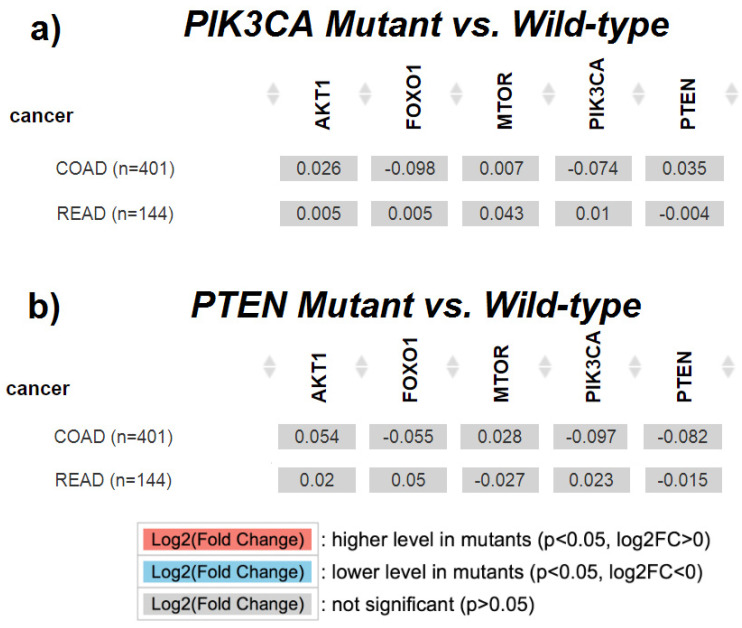
Comparison of gene expression with regard to the presence or absence of mutations. *p*-values were calculated using the Wilcoxon test; (**a**) *AKT1*, *FOXO1*, *mTOR* (*FRAP*), *PIK3CA*, and *PTEN* expressions with regard to *PIK3CA* gene mutation (Mutant vs. Wild-type); (**b**) *AKT1*, *FOXO1*, *mTOR* (*FRAP*), *PIK3CA*, and *PTEN* expressions with regard to *PTEN* gene mutation (Mutant vs. Wild-type).

For the analysis conducted using the TIMER 2.0 platform, the data from 401 colon adenocarcinoma (COAD) samples and 144 rectal adenocarcinoma (READ) samples were utilized. Mutations in the *PIK3CA* gene were present in 31.5% of COAD samples and 17.7% of READ samples. Mutations in the *PTEN* gene were much less frequent (5.9% in COAD and 5.3% in READ). The conducted statistical analysis did not show significant differences in the expression of *PIK3CA*, *PTEN*, *AKT1*, *FOXO1*, and *mTOR* (*FRAP*) genes with regard to the presence or absence of *PIK3CA* and *PTEN* gene mutations. The detailed results are presented in Figure 5.

### 2.6. The Association between PIK3CA, PTEN, AKT1, FRAP, FOXO1 Gene Expressions and Consensus Molecular Subtype (CMS)

**Figure 6:**
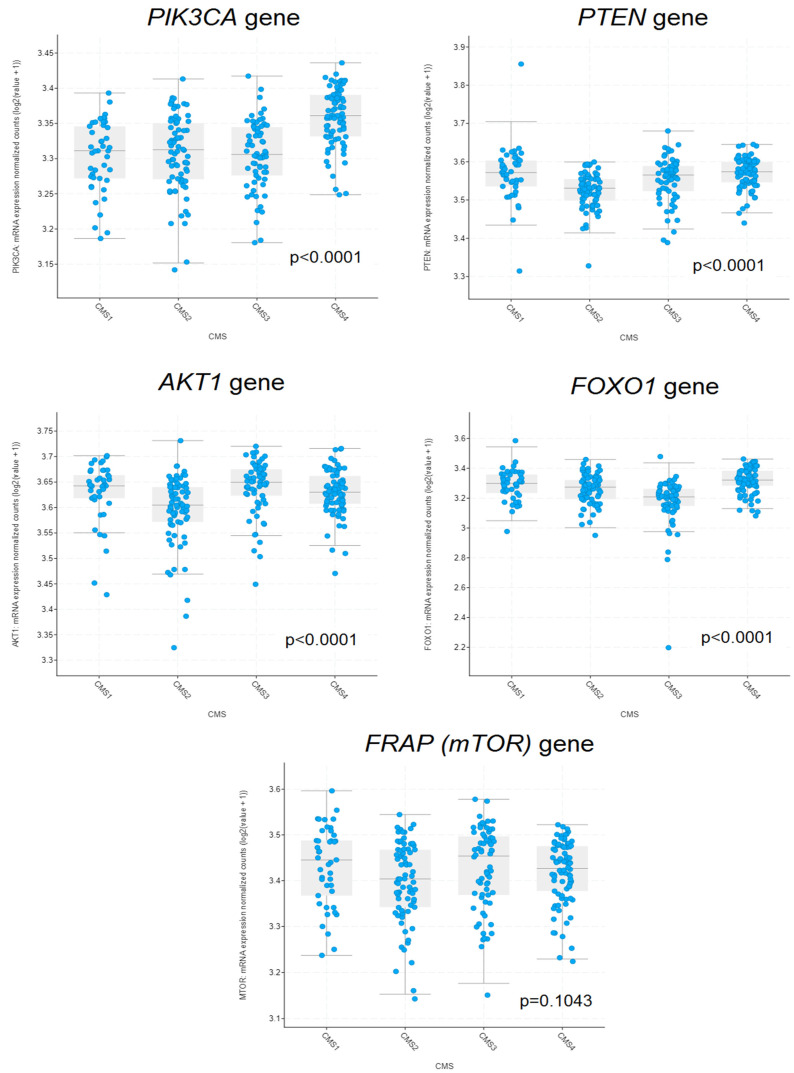
Differences in the expression of *PIK3CA*, *PTEN*, *AKT1*, *FOXO1*, and *FRAP* (*mTOR*) genes in colorectal cancer tissue according to CMS. *p*-values were calculated using one-way ANOVA.

Data from 267 CRC patients were used for the analysis. Within this group, 43 samples were classified as the MSI Immune subtype (CMS 1), 76 as the Canonical subtype (CMS 2), 66 as the Metabolic subtype (CMS 3), and 82 as the Mesenchymal subtype (CMS 4). A statistically significant relationship was demonstrated between CMS and the expression levels of the *PIK3CA* gene (*p* < 0.0001), *PTEN* (*p* < 0.0001), *AKT1* (*p* < 0.0001), and *FOXO1* (*p* < 0.0001). The expression level of the *FRAP* (*mTOR*) gene did not significantly differ depending on CMS (*p* = 0.1043) (Figure 6). The highest average expression values of the *PIK3CA* gene were observed in CMS 4. In the same subtype, the highest average values of the *FOXO1* gene were noted. Both *PTEN* and *AKT1* exhibited the lowest expression in the CMS 2 subtype (Figure 6).

### 2.7. Survival Analysis Based on the PIKCA, PTEN, AKT1, FRAP, and FOXO1 Gene Expressions in Patients with Colon Cancer

**Figure 7:**
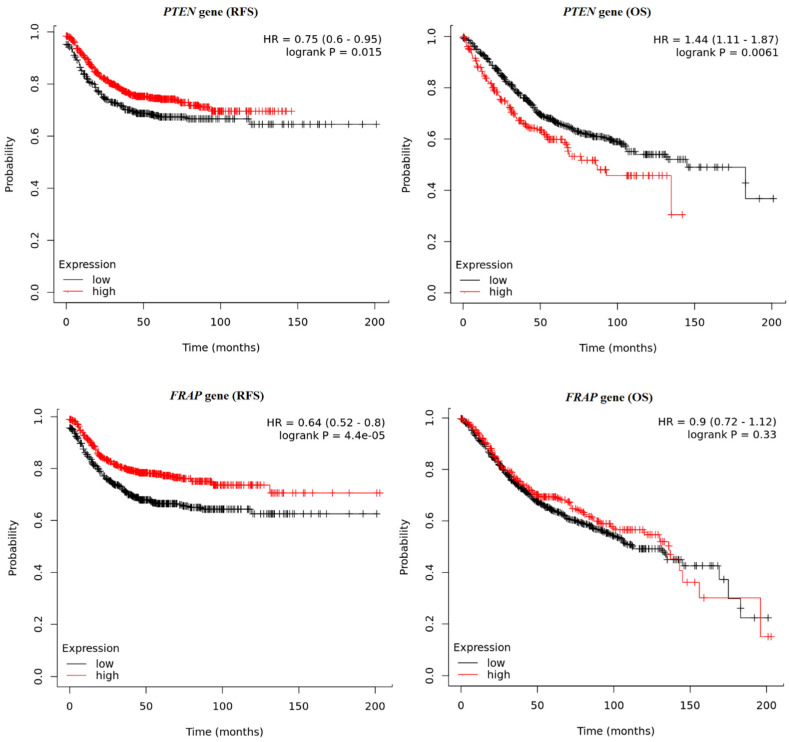
Kaplan–Meier curves representing the relapse-free survival (RFS) and overall survival (OS) of colon cancer patients based on the expression of the *PTEN* and *FRAP* genes.

**Figure 8:**
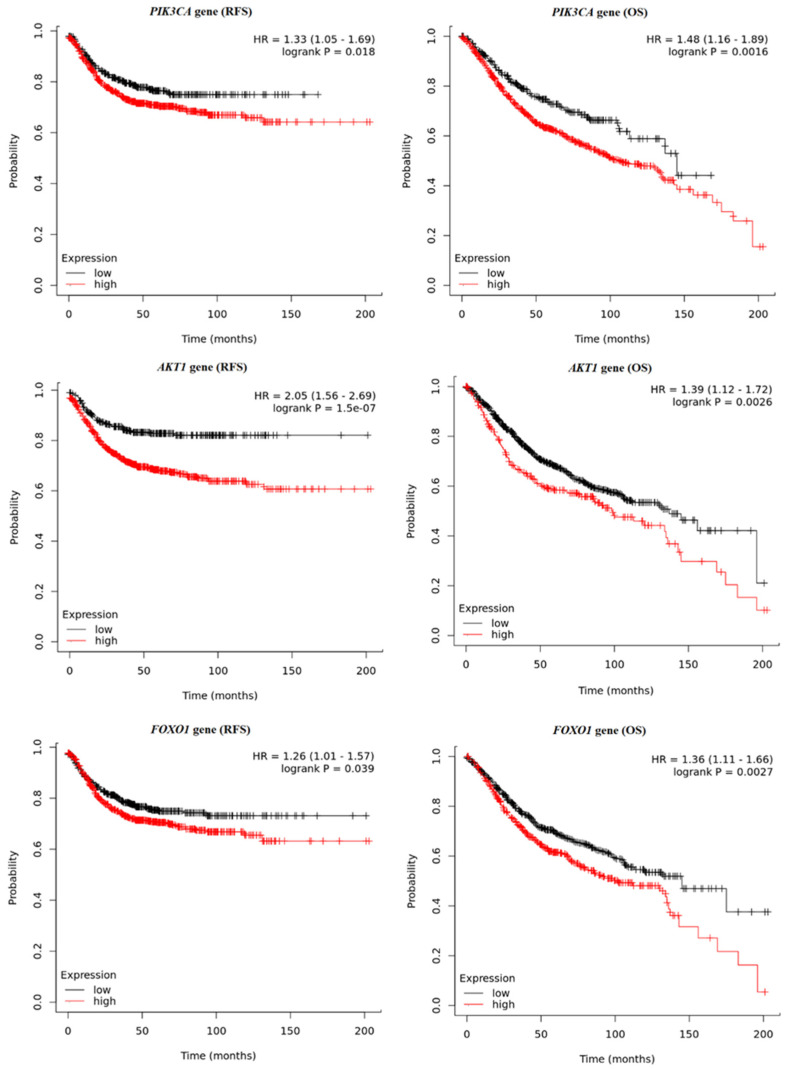
Kaplan–Meier curves representing the relapse-free survival (RFS) and overall survival (OS) of colon cancer patients based on the expression of the *PIK3CA*, *AKT1*, and *FOXO1* genes.

Briefly, the survival times of the colon cancer patients were compared with their gene expression pattern based on data obtained from the Kaplan–Meier plotter database. Significantly longer relapse-free survival (RFS) was observed among patients with higher *PTEN* (*p* = 0.0154) and *FRAP* expression (*p* < 0.0001). The detailed results are presented in Figure 7. The reverse results were observed for *PIK3CA* and *AKT1*; significantly longer RFS were noted for patients with lower *PIK3CA* (*p* = 0.0182) and *AKT1* (*p* < 0.0001). In the case of RFS analysis for the *FOXO1* gene, the obtained *p*-value (0.0391) was not statistically significant after performing multiple testing corrections (Figure 8). Overall survival (OS) analysis showed similar relationships for *PIK3CA* (*p* = 0.0016), *AKT1* (*p* = 0.0026), and *FOXO1* (*p* = 0.0027), where longer survival times were observed in the group of patients with higher expression of these genes (Figure 8). OS was not correlated with *FRAP* gene expression (*p* = 0.3326). However, for the *PTEN* gene, the analyses showed longer (OS) times for the group of patients with lower *PTEN* gene expression (*p* = 0.0061) (Figure 7).

## 3. Discussion

The PI3K/Akt pathway is frequently dysregulated in colorectal cancer (CRC), and CRC patients commonly demonstrate mutations in the *PIK3CA* gene, encoding the catalytic subunit of PI3K [12]. Such genetic alterations result in sustained activation of Akt, promoting cell survival and proliferation. Furthermore, the PI3K/Akt pathway can crosstalk with other signaling cascades, such as the Wnt/β-catenin pathway, further contributing to the malignant transformation of cells [13]. The activated PI3K/Akt pathway has been linked to several aspects of cancer progression, including increased resistance to apoptosis, enhanced angiogenesis, and promotion of metastasis [14]. It also participates in the regulation of the tumor microenvironment and the immune response, influencing the interaction that occurs between cancer cells and the surrounding tissues [15].

The mammalian target of rapamycin (mTOR) and Forkhead box protein O1 (FOXO1) are critical components of the cellular signaling pathway and appear to play a significant role in the process of carcinogenesis. mTOR is a central regulator of cell growth and protein synthesis. The protein is also involved in the regulation of angiogenesis. Increased angiogenesis is a hallmark of cancer, and mTOR over-activation can drive the initiation and progression of CRC [16,17]. FOXO1 is a transcription factor that plays a role in regulating cellular growth and apoptosis; its inactivation or loss of function in CRC can allow the evasion of such processes, allowing cancer cells to escape programmed cell death and continue proliferating [18].

The aim of the study was to determine the expression of key genes involved in the PI3K/AKT signaling pathway, including *PIK3CA*, *PTEN*, *AKT1*, *FOXO1*, and *FRAP*, in patients with CRC. The results were correlated with various clinicopathological and demographic characteristics: age, sex, tumor location, histological grade, disease stage, lymph node involvement, and the presence of angiogenesis and neuroinvasion.

It was found that changes in *FOXO1* gene expression depended on patient age, with higher expressions observed in the tumor tissue of patients above the median age of the studied group (68 years) compared to the younger group. Comparable results were obtained by Khan et al., who demonstrated higher *FOXO1* expression in breast cancer tissue of patients aged above 50 years compared to those under 50 years [19].

Higher *FOXO1* expression was also noted in tumor tissue originating from the rectum compared to tumors located within the colon. This may indicate that the expression of the analyzed gene varies depending on the specific part of the large intestine. Similarly, Ko et al. report significantly more frequent FOXO1 protein expression in tumor specimens originating from the rectum and the left side of the colon compared to the right side of the colon [20]. However, it is also possible that the genetic mechanisms underlying the process of carcinogenesis may vary between different parts of the large intestine.

Loss of PTEN function or downregulation of *PTEN* gene expression is significantly correlated with cancer incidence but also with a higher grade of malignancy and poorer prognosis in the case of various cancers, including colorectal cancer [21,22]. A team led by Yazdani et al. demonstrated that negative protein expression was associated with larger tumor size and higher cancer stage, and they identified a significant positive correlation between the mRNA level of the *PTEN* gene and PTEN protein expression [22]. In our study, no correlations were found between *PTEN* gene expression and features such as tumor size or cancer stage; however, higher *PTEN* gene expression was associated with a higher histological malignancy grade and a more frequent occurrence of neuroinvasion. These results differ from those noted in some previous studies [22,23]. Nevertheless, the relationship between the level of *PTEN* gene expression and parameters indicating the advancement of cancer may be more complex and depend on many additional factors. The level of *PTEN* gene expression may be independently influenced by the occurrence of genetic mutations or the overlap of epigenetic changes. Moreover, the simultaneous occurrence of other disorders in different signaling pathways in the course of carcinogenesis or the tumor microenvironment may influence changes in the expression of the *PTEN* gene. An additional individual factor is the therapeutic regimen used in patient treatments. Some targeted therapies in advanced colorectal cancer may indirectly influence the expression of the *PTEN* gene significantly through various mechanisms [24]. The complexity of processes influencing the level of gene expression may lead to ambiguous and different observations and relationships. In addition, it is also important to consider whether the changes involve mRNA or protein expression.

The bioinformatic analysis also compared the expression of the analyzed genes between normal and tumor tissues based on data from the UALCAN database. The analysis identified significantly higher *PTEN* and *FOXO1* expressions in normal tissue compared to tumor tissue in both the colon and rectum. Analysis of tissue images from HPA also confirmed higher levels of these proteins in normal colon and rectal tissue compared to tumor tissue. These results suggest that these genes may have a potential role in maintaining normal cellular function and suppressing carcinogenesis. This aligns with the existing data implicating *PTEN* and *FOXO1* in tumor suppression, and that their loss may contribute to cancer initiation and progression [21,22,23,25].

CRC is a very heterogeneous disease with diverse molecular backgrounds and various responses to treatment. Based on gene expression profiles, genomic and epigenetic changes, as well as clinical characteristics of patients, scientists have created a new molecular classification called Consensus Molecular Subtypes (CMS) [26]. In the conducted bioinformatics analysis, the gene expression levels of the PI3K/AKT pathway in CRC patients with defined CMS were used. The highest values of *PIK3CA* expression were observed in the mesenchymal subtype (CMS 4) which is associated with aggressive cancer development and poor prognosis [27]. Conversely, both *PTEN* and *AKT1* showed the lowest expression in the canonical molecular subtype (CMS 2). This is the most common molecular subtype among CRC patients and it is associated with good prognosis [28]. The obtained results indicate the presence of characteristic expression profiles of the PI3K/AKT pathway in different molecular subtypes of CRC, which can be utilized for molecular profiling.

Using the Kaplan–Meier plotter platform, the RFS and OS of the colon cancer patients were also analyzed based on the expression of the investigated genes. It was found that the high expression levels of *PTEN* and *FRAP* were correlated with better RFS rates. When the OS rate was assessed, no significant correlation was found with regard to the expression level of the *FRAP* gene, while for the *PTEN* gene, an inverse relationship was observed, where higher expression values correlated with worse survival outcomes. It has also been shown that both RFS and OS rates were lower for patients with higher expression values of the *PIK3CA* and *AKT1* genes. Da Costa et al. found low levels of PTEN protein expression to be associated with lower overall survival rates in head and neck squamous cell carcinoma patients treated with chemotherapy [29]. The opposite was noted in the case of advanced breast cancer, where high levels of *PTEN* gene expression were an unfavorable prognostic factor in the presence of functional TP53 protein [30]. The observed differences may have several causes. The studies being compared were conducted on different molecular levels in the groups of patients with different types of cancers. The most important potential factor is the use of various survival indicators, namely RFS, OS, and PFS (progression-free survival), which can provide different observations within the same group of patients. It can be seen that our findings, together with those in previous studies, indicate the need for further analysis in this area. It is also very important to clearly specify which indicator was used to assess survival time.

The conducted research also aimed to explore the diagnostic and clinical application of the obtained results in CRC patients. One of the possible applications of changes in the PI3K/Akt/mTOR pathway is to use them as prognostic markers. An increase in the activity of this pathway may be associated with a more aggressive form of CRC, which may have clinical application at the level of predicting the course of the disease and assessing the risk of recurrence [31,32]. Understanding changes in the activation of the PI3K/Akt pathway may enable the selection of personalized therapy for a specific group of patients. Patients with excessive activity of the PI3K/Akt/mTOR may be candidates for therapy with mTOR, Akt, and PI3K inhibitors to achieve greater treatment effectiveness. However, patients with low or missing PI3K/Akt/mTOR pathway activity may be resistant to treatment with those kinds of inhibitors [33]. In the bioinformatic analysis, we demonstrated correlations between the expression levels of genes associated with the PI3K/Akt pathway and the survival time of patients. This indicates the potential utility of such data as prognostic markers for CRC patients. However, further research is needed to confirm the clinical usefulness of the PI3K/Akt/mTOR pathway gene expression profile and the possibility of implementing them as markers in routine clinical practice.

We are aware that the above study has certain limitations. Firstly, we did not have access to normal tissue adjacent to the tumor; therefore, we could not perform a comparative analysis of the expression levels of selected genes between tumor tissue and histologically normal tissue. To compensate for this limitation, we conducted a bioinformatic analysis using publicly available data from online databases. Another limitation is the lack of data concerning the status of the *PIK3CA* and *PTEN* genes. In different types of cancer, *PIK3CA* gene mutations are often observed, which may influence both the activity level of the PI3K/Akt pathway and the expression profile of the analyzed genes.

## 4. Materials and Methods

### 4.1. Patients and Material

All patients enrolled in the study had received a diagnosis of colorectal cancer and were hospitalized in the Department of General and Colorectal Surgery (Medical University of Lodz, Poland) between January 2020 and June 2023. Among the 60 patients who qualified for the study, 31 were male and 29 were female. The median age of the patients was 68 years old (ranging from 34 to 90). The clinicopathological and demographic characteristics describing the study group are presented in Table 1. The samples of tumor tissue were collected intraoperatively during tumor removal surgery or colonoscopy. CRC tissues were immediately immersed in StayRNA™ (A&A Biotechnology, Gdańsk, Poland) preservation solution and frozen at −80 °C until isolation. A diagnosis of CRC was confirmed by pathologists based on histological examination. All colorectal cancer samples used in this study were classified as adenocarcinoma. The study was conducted in accordance with the principles of the Declaration of Helsinki and was approved by the Ethical Committee of the Medical University of Lodz (approval no. RNN/84/20/KE with amendments numbered KE/95/22 and KE/92/24). All research participants gave their signed, informed consent to take part.

### 4.2. RNA Extraction, Quantity, and Quality

RNA was extracted from neoplastic tissue using an RNeasy Mini Kit (Qiagen, Hilden, Germany) in accordance with the manufacturer’s protocol. The purity and concentration of the isolated RNA were assessed spectrophotometrically with a NanoPhotometer (Implen, Munich, Germany). RNA samples whose A260/A280 absorbance ratio was in the range of 1.8–2.0 were used for further steps. All samples were stored at −80 °C until further analysis.

### 4.3. Reverse Transcription Reaction

cDNA was obtained by reverse transcription (RT) using a High-Capacity cDNA Reverse Transcription Kit (Applied Biosystems, Waltham, MA, USA). Before performing RT, the RNA concentration in each sample was adjusted to 0.03 µg/µL, resulting in a final yield of 0.6 µg of RNA per sample. The reaction was carried out according to the manufacturer’s protocol. The obtained cDNA fragments were stored at −20 °C until further steps. To assess the correctness of this step, the *GAPDH* housekeeping gene was amplified using the REDTaq^®^ ReadyMix™ kit (Merck, Darmstadt, Germany). The following primers were used in the reaction: forward 5′-TGGTATCGTGGAAGGACTCATGAC-3′; reverse 5′-ATGCCAGTGAGCTTCCCGTTCAGC-3. The presence of a 96 base-pair product was checked using 2% agarose gel electrophoresis.

### 4.4. qPCR with Taqman Probes

The relative expressions of the *PIK3CA*, *PTEN*, *AKT1*, *FOXO1*, and *FRAP* genes were assessed in relation to two reference genes, *GAPDH* and *ACTB*, using the TaqMan™ Fast Advanced Master Mix kit (Thermofisher, Waltham, MA, USA). All reactions were performed on QuantStudio 5 Dx platform (Thermofisher, USA) under the following thermal conditions: initial denaturation at 95 °C for 10 min, followed by 40 cycles of denaturation at 95 °C for 15 s, and annealing/elongation at 60 °C for 60 s. The single reaction consisted of 10 µL of MasterMix, 2 µL of TaqMan probes and primers (1 µL of FAM-labeled probe and 1 µL of VIC-labeled probe), 7 µL of pure water, and 1 µL of previously obtained cDNA. Each sample was evaluated in duplicate. A negative control was performed for each series. The Taqman probes (Thermofisher, USA) used in the experiment are presented in Table 2. The means of the Ct values obtained from technical replicates were calculated for two reference and five investigated genes in each analyzed sample. The relative levels of the selected gene expression and the R values were determined by the ΔΔCt method due to the similar amplification efficiency for each gene [34]. The calibrator was calculated as the difference between the average Ct value of the target gene and the average Ct value of both reference genes, which were calculated from the entire study population.

### 4.5. Bioinformatics Analysis

The differences in the expression of the chosen genes between tumor tissue (colon adenocarcinoma and rectal adenocarcinoma) and normal tissue were determined based on data from The Cancer Genome Atlas (TCGA) RNA-seq available on the UALCAN website (http://ualcan.path.uab.edu/index.html, accessed on 20 November 2023) [35,36]. TIMER 2.0 (http://timer.cistrome.org/, accessed on 11 May 2024) was used to analyze the relationship between gene expression level and the occurrence of *PIK3CA* and *PTEN* gene mutations. Differential expression of each study gene is presented as log2 fold changes [37]. The assessment of the relationship between the molecular subtype of colorectal cancer and the expression levels of the genes analyzed in the study was conducted via the cBioPortal (http://cbioportal.org, accessed on 15 May 2024) based on data from the (Sidra-LUMC AC-ICAM dataset, Nat Med 2023) [38]. *p*-values were derived from one-way ANOVA. To compare the expression of the selected proteins of the PI3K/AKT pathway between tumor tissue and normal tissue, images of immunohistochemically stained specimens available in the Human Protein Atlas (HPA) (https://www.proteinatlas.org/, accessed on 9 May 2024) were used [39]. The Kaplan–Meier plotter (https://kmplot.com/analysis/, accessed on 24 November 2023) was used to compare RFS and OS between two groups of patients divided according to the expression of the analyzed genes. The option of automatic selection of the best cut-off was utilized to divide patients into a low-expression cohort and a high-expression cohort. The log-rank test was used to identify significant differences between the two groups [40].

### 4.6. Statistical Analysis of Experimental Data

Statistical analysis was conducted using the Statistica 13.1 software (TIBCO, Palo Alto, CA, USA). R-values were calculated after BOX-COX transformation. The normality of the distribution of any continuous variables was confirmed using the Shapiro–Wilk test. Any significant relationships between gene expression and the clinicopathological and demographic features among the CRC patients were determined using Student’s *t*-test, Kruskal–Wallis, Mann–Whitney U-test, correlation matrices, and ANOVA test, as appropriate. Benjamini-Hochberg method for multiple testing with an FDR of 0.1 was used where necessary. Results with a *p*-value below 0.05 were considered statistically significant. In the study, nominal *p*-values were provided; however, annotations were added if, after Benjamini-Hochberg correction, the dependencies lost statistical significance.

## 5. Conclusions

Our findings provide valuable insight into the complex role played by the genes of the PI3K/AKT pathway in CRC and are in line with those of previous studies indicating that the pathway components may act as biomarkers and therapeutic targets for the disease. Gene expression was found to correlate with clinicopathological parameters and survival outcomes; however, further investigation is needed to determine the functional implications of altered gene expression in CRC to improve our understanding of its pathogenesis and to improve patient management. Understanding the molecular intricacies of the PI3K/AKT pathway in CRC, especially their relationships with patient characteristics, may contribute to the development of enhanced personalized treatment strategies.

## Figures and Tables

**Table 1:** Characteristics of the study group.

Characteristic	Categories	n (%)
Sex	Male	31 (51.6)
	Female	29 (48.4)
Tumor localization	Rectum	29 (48.4)
	Sigmoid colon	13 (21.7)
	Ascending colon	5 (8.3)
	Rectosigmoid junction	4 (6.7)
	Splenic flexure	4 (6.7)
	Hepatic flexure	4 (6.7)
Tumor size (TNM)	Tis	1 (1.7)
	T2	16 (26.7)
	T3	30 (50.0)
	T4	9 (15.0)
	Missing	4 (6.7)
Lymph node status (TNM)	N0	33 (55.0)
	N1	11 (18.3)
	N2	12 (20.0)
	Missing	4 (6.7)
Distant Metastasis (TNM)	M0	50 (83.3)
	M1	6 (10.0)
	Missing	4 (6.7)
AJCC stage	I	16 (26.7)
	II	17 (28.3)
	III	17 (28.3)
	IV	6 (10.0)
	Missing	4 (6.7)
Tumor grading	Low-Grade	44 (73.3)
	High-Grade	14 (23.4)
	Missing	2 (3.3)
Angioinvasion	−	32 (53.3)
	+	24 (40.0)
	Missing	4 (6.7)
Neuroinvasion	−	43 (71.7)
	+	13 (21.6)
	Missing	4 (6.7)

**Table 2:** TaqMan probes and primers used in qPCR.

Gene Symbol	Assay ID	Dye
*GAPDH*	Hs02758991_g1	FAM
*ACTB*	Hs01060665_g1	VIC
*PIK3CA*	Hs00907957_m1	FAM
*PTEN*	Hs02621230_s1	FAM
*FOXO1*	Hs00231106_m1	VIC
*FRAP*	Hs00234508_m1	VIC

## Data Availability

The raw data and the analytic methods will be available upon request. To access protocols or datasets, contact rafal.swiechowski@umed.lodz.pl.
